# The clinically approved antiviral drug sofosbuvir inhibits Zika virus replication

**DOI:** 10.1038/srep40920

**Published:** 2017-01-18

**Authors:** Carolina Q. Sacramento, Gabrielle R. de Melo, Caroline S. de Freitas, Natasha Rocha, Lucas Villas Bôas Hoelz, Milene Miranda, Natalia Fintelman-Rodrigues, Andressa Marttorelli, André C. Ferreira, Giselle Barbosa-Lima, Juliana L. Abrantes, Yasmine Rangel Vieira, Mônica M. Bastos, Eduardo de Mello Volotão, Estevão Portela Nunes, Diogo A. Tschoeke, Luciana Leomil, Erick Correia Loiola, Pablo Trindade, Stevens K. Rehen, Fernando A. Bozza, Patrícia T. Bozza, Nubia Boechat, Fabiano L. Thompson, Ana M. B. de Filippis, Karin Brüning, Thiago Moreno L. Souza

**Affiliations:** 1 Laboratório de Imunofarmacologia, Instituto Oswaldo Cruz (IOC), Fundação Oswaldo Cruz (Fiocruz), Rio de Janeiro, RJ, Brazil; 2 Instituto Nacional de Infectologia (INI), Fiocruz, Rio de Janeiro, RJ, Brazil; 3 National Institute for Science and Technology on Innovation on Neglected Diseases (INCT/IDN), Center for Technological Development in Health (CDTS), Fiocruz, Rio de Janeiro, RJ, Brazil; 4 Instituto de Tecnologia de Fármacos (Farmanguinhos), Fiocruz, Rio de Janeiro, RJ, Brazil; 5 Laboratório de Vírus Respiratório e do Sarampo, IOC, Fiocruz, Rio de Janeiro, RJ, Brazil; 6 Instituto de Biologia, Universidade Federal do Rio de Janeiro (UFRJ), Rio de Janeiro, RJ, Brazil; 7 Instituto de Ciências Biomédicas, Universidade Federal do Rio de Janeiro, Rio de Janeiro, RJ, Brazil; 8 Laboratório de Virologia Comparada e Ambiental, IOC, Fiocruz, Rio de Janeiro, RJ, Brazil; 9 SAGE –COPPE, UFRJ, Rio de Janeiro, RJ, Brazil; 10 Núcleo em Ecologia e Desenvolvimento Sócio-Ambiental de Macaé (NUPEM), Universidade Federal do Rio de Janeiro, Macaé, Rio de Janeiro, Brazil; 11 D’Or Institute for Research and Education (IDOR), Rio de Janeiro, RJ, Brazil; 12 Laboratório de Flavivírus, IOC, Fiocruz, Rio de Janeiro, RJ, Brazil; 13 BMK Consortium: Blanver Farmoquímica Ltda; Microbiológica Química e FarmacêuticaLtda; Karin Bruning & Cia, Ltda, Brazil

## Abstract

Zika virus (ZIKV) is a member of the *Flaviviridae* family, along with other agents of clinical significance such as dengue (DENV) and hepatitis C (HCV) viruses. Since ZIKV causes neurological disorders during fetal development and in adulthood, antiviral drugs are necessary. Sofosbuvir is clinically approved for use against HCV and targets the protein that is most conserved among the members of the *Flaviviridae* family, the viral RNA polymerase. Indeed, we found that sofosbuvir inhibits ZIKV RNA polymerase, targeting conserved amino acid residues. Sofosbuvir inhibited ZIKV replication in different cellular systems, such as hepatoma (Huh-7) cells, neuroblastoma (SH-Sy5y) cells, neural stem cells (NSC) and brain organoids. In addition to the direct inhibition of the viral RNA polymerase, we observed that sofosbuvir also induced an increase in A-to-G mutations in the viral genome. Together, our data highlight a potential secondary use of sofosbuvir, an anti-HCV drug, against ZIKV.

Zika virus (ZIKV) is a member of the *Flaviviridae* family, which includes several agents of clinical significance, such as dengue (DENV), hepatitis C (HCV), West Nile (WNV) and Japanese encephalitis (JEV) viruses. This emerging pathogen is an enveloped positive-sense single-stranded RNA virus. Although ZIKV is an arthropod-borne virus (arbovirus) transmitted by mosquitos of the genus *Aedes*^1^, transmission through sexual contact has been described^2^. The perception that ZIKV causes a mild and self-limited infection^3^,^4^ has been jeopardized in recent years, with outbreaks in the Pacific Islands and the Americas^1^,^5^. For instance, ZIKV spread explosively across the Brazilian territory and to neighboring countries in 2015, infecting more than 4 million people^6^. As the ZIKV epidemic has scaled up, the virus has been associated with congenital malformations, including microcephaly, and a broad range of neurological disorders in adults, including Guillain-Barré syndrome (GBS)^7^,^8^. These morbidities associated with ZIKV infection led the World Health Organization (WHO) to declare the Zika outbreak to be a public health emergency of international concern.

Antiviral treatments against ZIKV are therefore necessary not only to mitigate ZIKV-associated morbidities but also to impair the chain of transmission. Some broad-spectrum antivirals, such as interferons (IFNs), ribavirin and favipiravir, are not suitable for use against ZIKV because they can be harmful to pregnant women^9^. Alternatively, others have studied the use of Food and Drug Administration (FDA)-approved small molecule drugs against ZIKV^10^,^11^,^12^,^13^,^14^,^15^. Overall, these drugs might exert their anti-ZIKV activity at least in part by interfering with the cellular pathways important for ZIKV replication^10^,^11^,^12^,^13^,^14^,^15^.

The gene encoding the viral RNA polymerase shows the highest degree of conservation among the members of the *Flaviviridae* family^16^, and the clinically approved anti-HCV drug sofosbuvir targets this protein. Sofosbuvir is an uridine nucleotide prodrug, which is triphosphorylated within cells to target the viral RNA polymerase^17^. Sofosbuvir is a class B FDA-approved drug. Moreover, Australia’s regulatory agency on drug administration, the Therapeutic and Goods Administration (TGA), categorizes sofosbuvir as class B1: “Drugs which have been taken by only a limited number of pregnant women and women of childbearing age, without an increase in the frequency of malformation or other direct or indirect harmful effects on the human fetus having been observed.” Altogether, this information motivated us to investigate whether the chemical structure of sofosbuvir possesses anti-ZIKV activity. In the interest of disseminating public health information, we disclosed a preprint of our data showing the anti-ZIKV activity of sofosbuvir^18^. In the present investigation, we further studied the pharmacology of sofosbuvir in neuronal and non-neuronal cell types. We observed a direct inhibition of the viral RNA polymerase and an increase in A-to-G mutations in the viral genome due to sofosbuvir treatment, highlighting a potential secondary use of sofosbuvir.

## Materials and Methods

### Reagents

The antiviral sofosbuvir (β-d-2′-deoxy-2′-α-fluoro-2′-β-C-methyluridine) was donated by the BMK Consortium: Blanver Farmoquímica Ltda; Microbiológica Química e Farmacêutica Ltda; Karin Bruning & Cia. Ltda, (Taboão da Serra, São Paulo, Brazil). Ribavirin was received as a donation from the Instituto de Tecnologia de Farmacos (Farmanguinhos, Fiocruz). Sofosbuvir triphosphate (STP) (β-d-2′-deoxy-2′-α-fluoro-2′-β-C-methyluridine triphosphate), ribavirin triphosphate (RTP) and AZT triphosphate (AZT-TP) were purchased (Codontech.org, CA and Sierra Bioresearch, AZ). Interferon-alpha was purchased from R&D Bioscience. All small molecule inhibitors were dissolved in 100% dimethylsulfoxide (DMSO) and subsequently diluted at least 10^4^-fold in culture or reaction medium before each assay. The final DMSO concentrations showed no cytotoxicity. The materials for cell culture were purchased from Thermo Scientific Life Sciences (Grand Island, NY), unless otherwise mentioned.

### Cells

Human neuroblastoma (SH-Sy5y; ATCC) and baby hamster kidney (BHK-21) cells were cultured in MEM:F-12 (1:1) and MEM, respectively. African green monkey kidney (Vero) and human hepatoma (Huh-7) cells were cultured in DMEM. *Aedes albopictus* cells (C6/36) were grown in L-15 medium supplemented with 0.3% tryptose phosphate broth, 0.75 g/L sodium bicarbonate, 1.4 mM glutamine, and nonessential amino acids. The culture medium of each cell type was supplemented with 10% fetal bovine serum (FBS; HyClone, Logan, Utah), 100 U/mL penicillin, and 100 μg/mL streptomycin^19^,^20^. The mammalian cells were incubated at 37 °C in 5% CO_2_, whereas the mosquito cells were maintained at 26 °C. Passages of the SH-sy5y cells included both adherent and non-adherent cells.

### Virus

ZIKV was isolated from a serum sample of a confirmed case from Rio de Janeiro, Brazil. This sample was received and diagnosed by the Reference Laboratory for Flavivirus, Fiocruz, Brazilian Ministry of Health, as part of the surveillance system against arboviruses^3^. Brazilian ZIKV was originally isolated in C6/36 cells, titered by plaque-forming assays and further passaged at a multiplicity of infection (MOI) of 0.01. The virus was passaged by inoculating C6/36 cells for 1 h at 26 °C. Next, the residual virus particles were removed by washing with phosphate-buffered saline (PBS), and the cells were cultured for an additional 9 days. After each period, the cells were lysed by freezing and thawing and centrifuged at 1,500 × *g* at 4 °C for 20 min to remove cellular debris.

ZIKV was purified between fractions of 50% and 20% sucrose. The sucrose gradients were made in 40 mL ultracentrifuge tubes (Ultra-clear; Beckman, Fullerton, CA) in PBS without Ca^++^ and Mg^++^ (pH 7.4). The tubes were allowed to stand for 2 h at room temperature. Up to 20 mL of virus was added to each tube and centrifuged in an SW 28 rotor (Beckman) at 10,000 rpm for 4 h at 4 °C. The fractions were collected and assayed for total protein and for virus-induced hemagglutination (HA) analysis using turkey red blood cells (Fitzgerald Industries International, North Acton, MA). The fractions displaying HA activity (≥16 UHA/50 μL) were pooled and dialyzed against PBS without Ca^++^ and Mg^++^ (pH 7.4) and 10% sucrose overnight at 4 °C. The virus pools were filtered through 0.22-μm membranes (Chemicon, Millipore, Bedford, NY). The infectious virus titers were determined by plaque assays in BHK-21 cells, and the virus was stored at −70 °C for further studies.

### Cytotoxicity assay

Monolayers of 10^4^ BHK-21, 5 × 10^4^ SH-Sy5y, 1.5 × 10^4^ Vero or 1.5 × 10^4^ Huh-7 cells in 96-well plates were treated for 5 days with various concentrations of sofosbuvir or ribavirin as a control. Then, 5 mg/ml 2,3-bis-(2-methoxy-4-nitro-5-sulfophenyl)-2*H*-tetrazolium-5-carboxanilide (XTT) in DMEM was added to the cells in the presence of 0.01% of N-methyl dibenzopyrazine methyl sulfate (PMS). After incubating for 4 h at 37 °C, the plates were read in a spectrophotometer at 492 nm and 620 nm^21^. The 50% cytotoxic concentration (CC_50_) was calculated by a non-linear regression analysis of the dose–response curves.

### Plaque-forming assay

Monolayers of BHK-21 cells in 6-well plates were exposed to different dilutions of the supernatant from the yield-reduction assays for 1 h at 37 °C. Whenever virus yields were extremely low, viral particles from the supernatants were concentrated through 80-kDa centrifugal exclusion filters (Merk Millipore, Darmstadt, Germany) prior to plaque-forming assay. Next, the cells were washed with PBS, and culture medium containing 1% FBS and 3% carboxymethylcellulose (Fluka) (overlay medium) was added to cells. After 5 days at 37 °C, the monolayers were fixed with 10% formaldehyde in PBS and stained with a 0.1% solution of crystal violet in 70% methanol, and the virus titers were calculated by scoring the plaque-forming units (PFU).

### Yield-reduction assay

Monolayers of 10^4^ BHK-21, 5 × 10^4^ SH-Sy5y, 1.5 × 10^4^ Vero or 1.5 × 10^4^ Huh-7 cells in 96-well plates were infected with ZIKV at the indicated MOIs for 1 h at 37 °C. The cells were washed with PBS to remove residual viruses, and various concentrations of sofosbuvir, or interferon-alpha as a positive control, in culture medium with 1% FBS were added. After 24 h, the cells were lysed, the cellular debris was cleared by centrifugation, and the virus titers in the supernatant were determined as PFU/mL. A non-linear regression analysis of the dose-response curves was performed to calculate the concentration at which each drug inhibited the plaque-forming activity of ZIKV by 50% (EC_50_).

### Preparation of ZIKV RNA polymerase

ZIKV RNA polymerase (ZVRP) was obtained from ZIKV-infected BHK-21 cells. The cells were infected with ZIKV at an MOI of 10 for 24 h, lysed with buffer A [containing 0.25 M potassium phosphate (pH 7.5), 10 mM 2-mercaptoethanol (2-ME), 1 mM EDTA, 0.5% Triton X-100, 0.5 mM phenylmethane sulfonyl fluoride (PMSF) and 20% glycerol], sonicated and centrifuged at 10,000 × *g* for 10 min at 4 °C. The resulting supernatant was further centrifuged at 100,000 × *g* for 90 min at 4 °C and passed through two ion-exchange columns, DEAE- and phospho-cellulose^19^. Alternatively, the ZIKV NS5 region encoding the nucleotides responsible for the RNA-dependent RNA polymerase (RDRP) activity were cloned into the pET-41b+ vector (Novagen) between the BamHI and SacI sites. ZVRP expression was induced by adding isopropyl β-D-1-thiogalactopyranoside (IPTG) to the *E. coli* strain BL21. The cells were lysed in buffer A, and the N-terminal GST-tag was used to purify the protein using a GST spin purification kit (ThermoFisher Scientific) according to the manufacturer’s instructions.

### RNA polymerase inhibition assay

ZVRP inhibition assays were adapted from a previous publication^22^. The reaction mixture for ZVRP activity measurements was composed of 50 mM HEPES (pH 7.3), 0.4 mM of each ribonucleotide (ATP, GTP, CTP and labelled UTP), 0.4 mM dithiothreitol, 3 mM MgCl_2_, and 500 ng of ZIKV genomic RNA or cell extracts. The ZIKV RNA was obtained using a QIAmp viral RNA mini kit (Qiagen, Dusseldorf, Germany) according to the manufacturer’s instructions, except for the use of the RNA carrier. The reaction mixtures were incubated for 1 h at 30 °C in the presence or absence of the drugs. The reactions were stopped with addition of EDTA at a final concentration of 10 mM.

The labeled UTP mentioned above represents an equimolar ratio between biotinylated-UTP and digoxigenin-UTP (DIG-UTP) (both from Roche Life Sciences, Basel, Switzerland). The detection of incorporated labeled UTP nucleotides was performed by an amplified luminescent proximity homogeneous assay (ALPHA; PerkinElmer, Waltham, MA). In brief, streptavidin-donor and anti-DIG-acceptor beads were incubated with the stopped reaction mixture for 2 h at room temperature. Then, the plates containing the mixtures were read in an EnSpire^®^ multimode plate reader (PerkinElmer). Different types of blank controls were used, such as reaction mixtures without cellular extracts and a control reaction mixture without inhibitor and beads. In addition, the extract from mock-infected cells was also assayed to evaluate the presence of RNA-dependent RNA polymerase activity unrelated to ZIKV. Non-linear regression curves were generated to calculate the IC_50_ values for the dose-response effects of the compounds.

### Antiviral activity in human induced pluripotent stem (iPS) cell-derived neural stem cells (NSCs) and brain organoids

NSCs and brain organoids derived from human iPS cells were prepared as previously described^23^. The NSCs (20 × 10^3^ cells/well in a 96-well plate) were infected at MOIs of either 1.0 or 10 for 2 h at 37 °C. Next, the cells were washed, and fresh medium containing sofosbuvir was added. The cells were treated daily with sofosbuvir at the indicated concentrations. The NSCs were observed daily for 8 days after infection. Virus titers were determined from the culture supernatant using a plaque-forming assay. The cell supernatant was also used for ZIKV genome analysis. Cell death was measured by adding 2 μM CellEvent caspase-3/7 reagent and the fluorescent dye ethidium homodimer^23^ at days 4 and 8, when the culture supernatants were collected. Images were acquired with an Operetta high-content imaging system with a 20x objective and high numerical apertures (NA) (PerkinElmer, USA). The data were analyzed using the high-content image analysis software Harmony 5.1 (PerkinElmer, USA). Seven independent fields were evaluated from triplicate wells per experimental condition.

Brain organoids were infected with ZIKV at 3 × 10^5^ PFU/mL for 2 h, and the medium containing virus particles was then replaced with fresh medium. Sofosbuvir was added to the fresh medium daily for one week. The culture supernatant was collected to monitor virus infectivity and for RNA sequencing.

### Comparative molecular modeling

The amino acid sequence encoding ZVRP (UniProtKB ID: B3U3M3) was obtained from the EXPASY proteomic portal^24^ (http://ca.expasy.org/). The template search was performed using the Blast server (http://blast.ncbi.nlm.nih.gov/Blast.cgi) with the Protein Data Bank^25^ (PDB; http://www.pdb.org/pdb/home/home.do) as the database and the default options. The T-COFFEE algorithm was used to generate a multiple alignment between the amino acid sequences of the template proteins and ZVRP. Subsequently, the construction of the SFV-ZVRP complex was performed using MODELLER 9.16 software^26^, which employs spatial restriction techniques based on the 3D-template structure. The preliminary model was refined in the same software, using three cycles of the default optimization protocol. The structural evaluation of the model was then performed using two independent algorithms in the SAVES server (http://nihserver.mbi.ucla.edu/SAVES_3/): PROCHECK software^27^ (stereochemical quality analysis) and VERIFY 3D^28^ (compatibility analysis between the 3D model and its own amino acid sequence by assigning a structural class based on its location and environment and by comparing the results with those of crystal structures).

### Genome assembly

A 0.3-mL aliquot of supernatant containing ZIKV (at least 2 × 10^5^ PFU) was filtered through 0.22-μm filters to remove residual cells. The viral RNA was extracted using a QIAamp Viral RNA Mini Kit (Qiagen^®^) with RNase-free DNase (Qiagen^®^) treatment, omitting carrier RNA. Double-stranded cDNA libraries were constructed using a TruSeq Stranded Total RNA LT kit (Illumina^®^) with Ribo-zero treatment according to the manufacturer’s instructions. The library size distribution was assessed using a 2100 Bioanalyzer (Agilent^®^) with a High Sensitivity DNA kit (Agilent^®^), and the quantification was performed using a 7500 Real-time PCR System (Applied Biosystems^®^) with a KAPA Library Quantification Kit (Kapa Biosystems). Paired-end sequencing (2 × 300 bp) was performed with a MiSeq Reagent kit v3 (Illumina^®^). The sequences obtained were preprocessed using the PRINSEQ software to remove reads smaller than 50 bp and sequences with scores of lower quality than a Phred quality score of 20. Paired-End reAd merger (PEAR) software was used to merge and extend the paired-end Illumina reads using the default parameters^29^,^30^. The extended reads were analyzed against the Human Genome Database using the DeconSeq program, with an identity and coverage cutoff of 70%, to remove human RNA sequences^31^. Non-human reads were analyzed against all GenBank viral genomes (65 052 sequences) using the BLAST software with a 1e-5 e-value cutoff. The sequences rendering a genome were assembled with SPAdes 3.7.1 software^32^ followed by a reassembly with the CAP3 program^33^.

### Sequence comparisons

The sequences encoding the C-terminal portion of the RNA polymerase from members of the *Flaviviridae* family were acquired from the complete sequences deposited in GenBank. An alignment was performed using the ClustalW algorithm in the Mega 6.0 software. The sequences were analyzed using the neighbor-joining method with pairwise deletion and a bootstrap of 1,000 replicates, and the *P* distances were registered. The sequences were also analyzed for the mean evolutionary rate.

### Statistical analysis

All assays were performed and codified by one professional. Subsequently, a different professional analyzed the results before the identification of the experimental groups. This approach was used to keep the pharmacological assays blind. All experiments were carried out at least three independent times, including technical replicates in each assay. The dose-response curves used to calculate the EC_50_ and CC_50_ values were generated by Excel for Windows. The dose-response curves used to calculate the IC_50_ values were produced by Prism GraphPad software 5.0. The equations to fit the best curve were generated based on R^2^ values ≥ 0.9. ANOVA tests were also used, with *P* values < 0.05 considered statistically significant. The statistical analyses specific to each software program used in the bioinformatics analysis are described above.

## Results

### Sofosbuvir fits into the ZVRP predicted structure

The RNA polymerase structures from WNV (PDB #2HFZ)^34^, JEV (PDB #4K6M)^35^, DENV (PDB #5DTO)^36^ and HCV (PDB #4WTG)^37^ share 72, 70, 68, and 25% sequence identity, respectively, with the orthologous ZIKV enzyme. Despite its relatively low sequence identity to ZIKV, the HCV enzyme structure is complexed with sofosbuvir, and the amino acids residues that interact with the drug are highly conserved (approximately 80%) among the members of the *Flaviviridae* family^37^. The region encoding the C-terminal portion of the *Flaviviridae* RNA polymerase contains around 800 amino acid residues. Of these, we have highlighted in yellow those that are identical among members of the *Flaviviridae* family (see Supplementary Material 1). The residues critical for RDRP activity are conserved among different viral species and strains, including an African ZIKV strain from the 1950 s and those circulating currently, DENV and different genotypes of HCV (see Supplementary Material 1)^38^.

**Figure 1:**
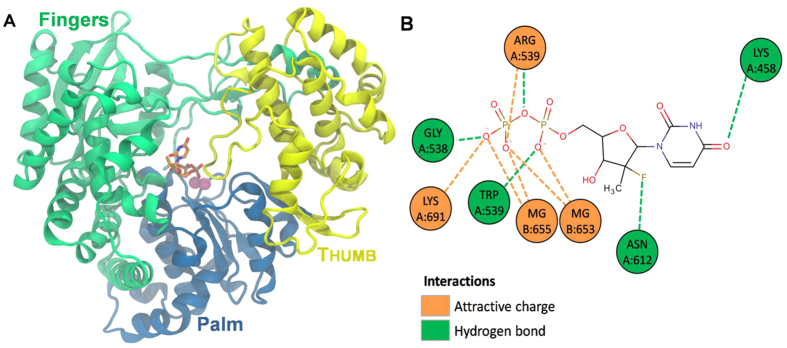
Putative ZIKV RNA polymerase in complex with sofosbuvir. Based on the crystal structure of the HCV RNA polymerase in complex with sofosbuvir diphosphate (PDB accession #4WTG), the putative structure of the ZVRP was constructed. Using the T-COFFEE server, the amino acid sequence of the ZVRP (UniProtKB ID: B3U3M3) was aligned with orthologous RNA polymerases from other members of the *Flaviviridae* family, specifically hepatitis C virus (HCV; PDB #4WTG, West Nile virus (WNIV; PDB #2HFZ), Japanese encephalitis virus (JEV; PDB #4K6M), and dengue virus (DENV; PDB #5DTO). The MODELLER 9.16 software was used to build a 3D model of ZIKV RNA polymerase, with subsequent refinement performed using three cycles of the default optimization protocol. The structural evaluation of the model was performed using two independent algorithms, PROCHECK software and VERIFY 3D. (**A**) The 3D model of ZIKV RNA polymerase is presented. (**B**) The residues presumably required for the interaction of ZVRP with sofosbuvir and Mg^++^ ions.

Based on the HCV RNA-dependent RNA polymerase domains, we constructed a 3D model of the orthologous ZIKV enzyme (Fig. 1). Sofosbuvir was predicted to be located among the palm and fingers region of ZIKV RNA polymerase (Fig. 1A), an area important for coordinating the incorporation of incoming nucleotides into the new strand of RNA^37^. Consequently, the amino acid residues relevant to the sofosbuvir interaction are some of those critical for natural nucleotide incorporation and thus RDRP activity (Fig. 1B)^37^.

### Sofosbuvir inhibits ZVRP in a dose-dependent fashion

**Figure 2:**
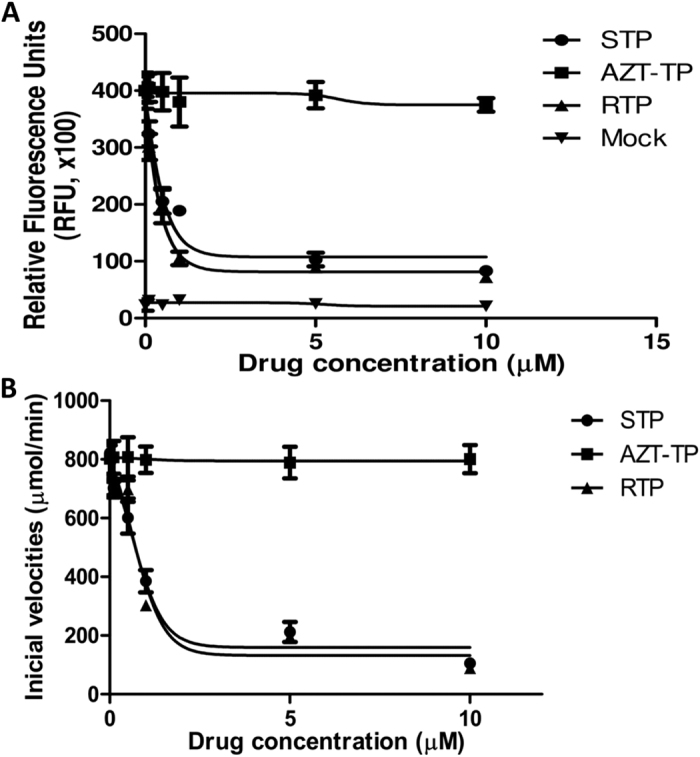
Sofosbuvir inhibits ZIKV RDRP activity. Cell extracts from ZIKV-infected cells (**A**) or recombinant ZVRP (**B**) were assayed for RDRP activity using viral RNA as the template and labeled UTP as the tracer. Biotinylated-UTP and digoxigenin-UTP were detected by ALPHA technology using an EnSpire^®^ multimode plate reader (PerkinElmer). The molecules assayed were sofosbuvir triphosphate (STP), ribavirin triphosphate (RTP) and AZT triphosphate (AZT-TP). As a control, the RDRP activity was measured in extracts from mock-infected cells (mock). The data represent means ± SEM of five independent experiments.

Next, we evaluated whether sofosbuvir triphosphate (STP), the bioactive compound, could inhibit ZIKV RDRP activity. Fractions containing the ZIKV RDRP activity were partially purified from infected cells^19^. STP inhibited ZIKV RDRP activity with an IC_50_ value of 0.38 ± 0.03 μM (Fig. 2A). Ribavirin-triphosphate (RTP) and AZT-TP were used as positive and negative controls, respectively (Fig. 2A). RTP and AZT-TP exhibited IC_50_ values of 0.21 ± 0.06 and >10 μM, respectively (Fig. 2A). Moreover, the recombinant expression of the C-terminal portion of ZVRP confirmed the STP antiviral activity, with an IC_50_ value of 0.61 ± 0.08 μM (Fig. 2B). The AZT-TP and RTP IC_50_ values for recombinant ZVRP were >10 and 0.62 ± 0.05 μM, respectively (Fig. 2B). Of note, the small discrepancy observed in the STP IC_50_ values against the partially purified and recombinant ZVRP could be because the total protein content was used to normalize the assay conditions; therefore, the purified recombinant preparation possessed a higher specific activity. Altogether, the data from Fig. 2 confirmed the molecular modeling prediction that sofosbuvir docks onto the ZVRP structure, revealing that the chemical structure of sofosbuvir inhibits ZIKV RDRP activity.

### Sofosbuvir inhibits ZIKV replication in a cell lineage-, MOI- and dose-dependent manner

Before investigating ZIKV susceptibility to sofosbuvir using cell-based assays, a Brazilian ZIKV isolate was characterized for further use as a reference strain for the experimental virology. The full-length viral genome was sequenced (GenBank accession #KX197205), and the characteristic plaque-forming units (PFU) and cytopathic effects (CPE) were determined in BHK-21 cells (see Supplementary Fig. S1). Another concern was to establish whether another plaque-forming viral agent was co-isolated with ZIKV, which could result in a misleading interpretation of the antiviral activity. A metagenomic analysis revealed that ZIKV was the only full-length genome of a plaque-forming virus detected in BHK-21 cells (see Supplementary Material 2).

**Figure 3:**
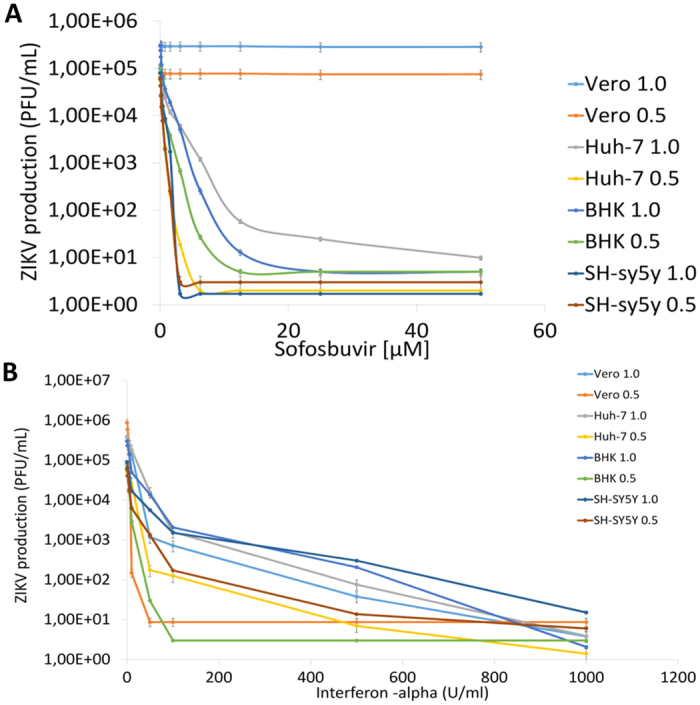
The antiviral activity of sofosbuvir against ZIKV. BHK-21, SH-sy5y, Huh-7 or Vero cells were infected with ZIKV at the indicated MOIs and exposed to various concentrations of sofosbuvir (**A**) or IFN-alpha (**B**), and the viral replication was measured by plaque-forming assays after 24 h of infection. The data represent means ± SEM of three independent experiments.

The sofosbuvir phosphoramidate prodrug must be converted to its triphosphate analog in the cellular environment to become active. Despite a general perception that this process is an exclusive feature of hepatocytes^17^, sofosbuvir may also become active within neuroepithelial stem cells^39^. Indeed we investigated whether sofosbuvir inhibits ZIKV replication in different cellular systems. BHK-21, SH-Sy5y, Huh-7 or Vero cells were inoculated at different MOIs and treated with various concentrations of sofosbuvir. The supernatants from these cells were collected, and the infectious virus progeny were titered. Sofosbuvir induced an MOI- and dose-dependent inhibition of ZIKV replication (Fig. 3A, Tables 1 and 2, and see Supplementary Fig. S2A–D). The potency and efficiency to inhibit ZIKV replication were higher in Huh-7 and SH-Sy5y cellsthan in BHK-21 cells (Fig. 3A and Table 1 and 2, and see Supplementary Fig. S2A–C). Of note, even high concentrations of sofosbuvir did not inhibit ZIKV replication in Vero cells, indicating a cell-dependent inhibition of ZIKV replication (Fig. 3A and Tables 1 and 2, and see Supplementary Fig. S2D). IFN-alpha and ribavirin were used as positive controls to inhibit ZIKV replication (Fig. 3B and Tables 1 and 2, and see Supplementary Fig. S2A–D).

The sofosbuvir cytotoxicity was also cell type-dependent (Tables 1 and 2). Our results indicate that the selectivity index (SI; which represents the ratio between the CC_50_ and EC_50_ values) for sofosbuvir varied from 184 to 1191 (Tables 1 and 2) – being safer at an MOI of 0.5 in the hepatoma and neuroblastoma cell lines. For comparison, the SI values for sofosbuvir were almost 30 times higher than for ribavirin (Table 1). Our data indicate that the sofosbuvir chemical structure possesses anti-ZIKV activity.

### Sofosbuvir inhibits ZIKV replication in human primary NSCs and brain organoids

**Figure 4:**
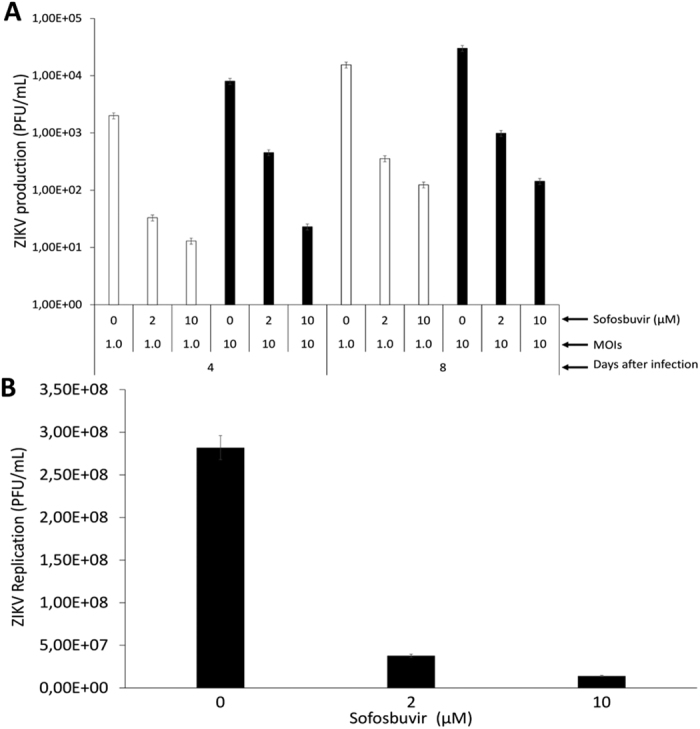
Sofosbuvir inhibits ZIKV replication in human iPS cell-derived NSCs and brain organoids. NSCs (**A**) were infected at the indicated MOIs and brain organoids (**B**) were infected with 5 × 10^7^ PFU/mL of ZIKV. The ZIKV-infected cells were treated with the indicated concentrations of sofosbuvir for different periods of time post infection. At the indicated time points, the culture supernatants were collected, and the virus was titered by plaque-forming assays. The data represent means ± SEM of five independent experiments. The virus production in the presence of the treatments was significantly reduced when compared to untreated cells (*P* < 0.01).

**Figure 5:**
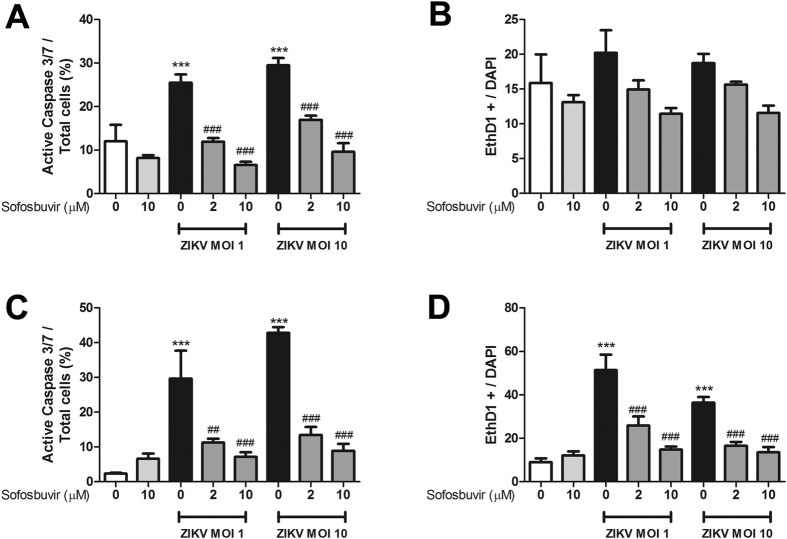
Sofosbuvir protects human iPS cell-derived NSCs from ZIKV-induced cell death. NSCs were labeled for activated caspase-3/7 (**A,C**) and cell permeability (**B,D**) 4 or 8 days after infection. The data represent means ± SEM of five independent experiments. ***indicates *P* < 0.001 for the comparison between the mock- and ZIKV-infected cells. ^###^Indicates *P* < 0.001 and ^##^Indicates P < 0.01 for the comparison between the ZIKV-infected cells treated with or without sofosbuvir.

Since the results regarding the pharmacologic activity of sofosbuvir against ZIKV replication in lineage cells were promising, we next investigated whether sofosbuvir could be neuroprotective in a cellular model that corresponds to the early stages of brain development^23^. Human iPS cell-derived neural stem cells (NSCs) were infected with ZIKV and treated with sofosbuvir. Sofosbuvir produced a pronounced inhibition of ZIKV replication in NSCs challenged with MOIs of 1.0 or 10 after 4 to 8 days of infection (Fig. 4A), as only marginal virus titers were detected in sofosbuvir-treated cells. This drastic reduction in viral replication in sofosbuvir-treated NSCs impaired ZIKV-mediated neuropathogenesis by inducing cell death. Whereas ZIKV-infected NSCs exhibited considerable levels of caspase-3/7 activation and plasma membrane permeability at 8 days post infection, sofosbuvir significantly protected these cells from death (Fig. 5, and see Supplementary Figs S3 and S4).

Subsequently, we used ZIKV-infected brain organoids as a three-dimensional model to assess the antiviral activity of sofosbuvir. Brain organoids have been used as a sophisticated cellular model to evaluate the impact of ZIKV on early brain development and as a translational system for in-depth investigations of the cellular and molecular events related to microcephaly^23^. We again observed a pronounced reduction of ZIKV production due to sofosbuvir treatment (Fig. 4B). Altogether, these data indicate that sofosbuvir is effective against ZIKV in neuronal cell systems relevant to the physiopathology of the virus.

### Observational analysis of mutations in the ZIKV genome associated with sofosbuvir treatment

**Figure 6:**
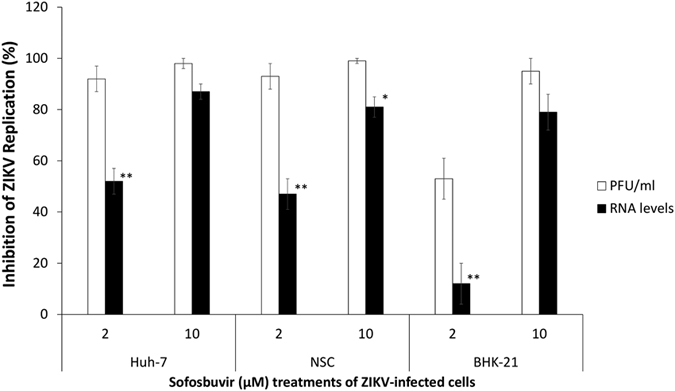
Inhibition of ZIKV-related infectivity and RNA production by sofosbuvir. ZIKV-infected Huh-7 cells, NSCs and BHK-21 cells were treated with or without sofosbuvir. The culture supernatants were collected 24 h after infection to determine the virus infectivity by plaque-forming assays and the viral RNA loads by real time RT-PCR analysis. The data are presented as the percentage over the control (untreated cells). *Indicates a significant difference (*P* < 0.05) between the black and white bars. **Indicates a significant difference (*P* < 0.01) between the black and white bars.

Systematically, we observed that sofosbuvir was more effective in reducing ZIKV infectivity than viral RNA levels in the supernatant of the cultures (Fig. 6). Independent of whether sofosbuvir activity was measured in ZIKV-infected Huh-7 cells, NSCs or BHK-21 cells, a concentration of 2 μM of this drug was in general almost two times more potent in inhibiting ZIKV-induced cell death and viral infectivity than viral RNA production (Fig. 6). These data suggest that virus particles containing genomic RNA may be produced under sofosbuvir treatment but that these particles are unable to efficiently infect new cells and produce cytopathogenicity.

We thus sequenced the ZIKV genome from infected Huh-7 cells, NSCs and brain organoids treated with sofosbuvir at 2 μM, a concentration that does not allow for viral RNA extinction. The frequencies of the overall transition mutations in sofosbuvir-treated sequences was significantly increased when compared to control sequences (*P* < 0.05) for all infected host cell models (Table 3). More specifically, the increased mutation frequency was greater for A-to-G changes, which represented 80 ± 5% of all transition mutations in the sofosbuvir-treated sequences (Table 3). In addition, A-to-G mutations were approximately five times more abundant in sofosbuvir-treated sequences than in control sequences (Table 3). Of note, there were no significant differences in the frequencies of other transitions and transversions between sofosbuvir-treated and control sequences. Our observations indicate that in addition to its direct inhibition of the ZIKV RNA polymerase, sofosbuvir may also increase mutations in the viral genome.

## Discussion

ZIKV is a member of the *Flaviviridae* family, along with other clinically relevant viruses such as DENV, WNV, JEV and HCV. In this family, ZIKV was considered to be a virus causing only mild and self-limited infections^4^. However, based on clinical evidence and experimental data, ZIKV infection has been associated with neurological-related morbidities, with impacts on the development of the human nervous system and neurological complications in adults^7^,^8^,^23^,^40^,^41^,^42^,^43^. Antiviral treatment options are thus required to block viral replication. Here, we show that uridine nucleotide analog anti-HCV drug sofosbuvir possesses anti-ZIKV activity.

Several ongoing studies have demonstrated the anti-ZIKV activity of clinically approved drugs^10^,^11^,^12^,^13^,^14^,^15^,^18^. Here, we show that sofosbuvir-triphosphate inhibits ZVRP in a dose-dependent fashion. The predicted ZVRP structure suggests that sofosbuvir binds to amino acid residues that are critical for ribonucleotide incorporation, such as Arg473, Gly538, Trp539, and Lys691. The fluoride radical in the sofosbuvir ribosyl moiety is coordinated by Asn612, an interaction that is involved with the drug selectivity to the viral RDRP, which may avoid nonspecific effects towards the cellular DNA-dependent RNA polymerase. Lys458 seems to be the docking residue for the uridine analog.

Next, using cell-based assays, we demonstrated that sofosbuvir inhibits ZIKV replication in BHK-21, SH-Sy5y and Huh-7 cells. Although ZIKV replication was susceptible to sofosbuvir in these cells, the magnitude of antiviral potency and efficiency varied among these cells. Taking as a reference an MOI of 1.0, the potency of antiviral activity observed in hepatoma cells was five times higher, and in neuroblastoma cells was two times higher, than that observed in BHK-21 cells. This is consistent with the demonstration that sofosbuvir converts to its bioactive form in liver cells^17^. Most recently, another group also demonstrated that sofosbuvir may become active in human neuroepithelial stem cells using functional assays against ZIKV^39^. Indeed, we further confirmed that human iPS cell-derived NSCs and brain organoids can be protected by sofosbuvir, even when these cells are challenged with exceedingly high MOIs. Brain organoids represent a translational three-dimensional cell culture model for the study of ZIKV-associated microcephaly^23^. Therefore, the antiviral activity observed by sofosbuvir using human neural progenitors and brain organoids represents a pragmatic demonstration of the potential secondary use of this clinically approved anti-HCV drug, because it impairs ZIKV neuropathogenesis, as shown here and by others^39^.

On the other hand, we observed no inhibition of viral replication with even 50 μM of sofosbuvir in Vero cells. Similarly, in a recent study from Eyer *et al*.^12^, African ZIKV susceptibility to sofosbuvir was screened in Vero cells, and this compound did not emerge as a potential hit. Although Eyer *et al*.^12^ and our group used different viral strains, we obtained similar results in Vero cells. Interestingly, sofosbuvir is a substrate for glycoprotein-P^44^. Proteomic data indicate that Vero cells express this multi-drug resistance ABC-transporter, which may cause the efflux of sofosbuvir from the cell^45^,^46^,^47^.

Using different read outs to monitor sofosbuvir anti-ZIKV activity, we observed that this drug reduced virus infectivity more than it inhibited the production of viral RNA. Unlike most antiviral nucleoside analogs^48^ and despite the presence of the 3′-OH radical in its chemical structure^37^, sofosbuvir acts as a chain terminator. To impair the incorporation of the incoming nucleotides, the 2′-F radical disrupts the hydrogen bonding network^37^. Similarly, the 2′-F in sofosbuvir forms a hydrogen bond with Asn612, which may disrupt the hydrogen bonding of incoming nucleotides to ZIKV RNA polymerase. Indeed, our results indicate that sofosbuvir directly inhibits the viral RNA polymerase. In addition to this mechanism, ZIKV genome sequences from sofosbuvir-treated cells had an increased rate of A-to-G mutations when compared to untreated cells. It is plausible to interpret that both the direct inhibition of ZIKV RNA polymerase and induction of mutations in the viral genome may be triggered by sofosbuvir. For instance, ribavirin, another ribonucleoside analog, may inhibit viral replication by direct targeting the viral RNA polymerase and by the induction of error-prone replication^49^,^50^.

ZIKV-associated microcephaly and GBS highlight that antiviral interventions are urgently needed. Our data reveal that a clinically approved drug possesses antiviral activity against ZIKV and is active in cells derived from peripheral organs and the CNS. Sofosbuvir may induce error-prone ZIKV replication. Together, our data highlight the potential secondary use of sofosbuvir, an anti-HCV drug, against ZIKV.

## Additional Information

**How to cite this article**: Sacramento, C. Q. *et al*. The clinically approved antiviral drug sofosbuvir inhibits Zika virus replication. *Sci. Rep.* **7**, 40920; doi: 10.1038/srep40920 (2017).

**Publisher's note:** Springer Nature remains neutral with regard to jurisdictional claims in published maps and institutional affiliations.

## Supplementary Material

Supplementary Information

Supplementary Material 1

Supplementary Material 2

## Figures and Tables

**Table 1:** Antiviral activity and cytotoxicity of sofosbuvir at an MOI of 1.0.

	Sofosbuvir			Ribavirin			IFN-alpha*		
	EC_50_	CC_50_	SI	EC_50_	CC_50_	SI	EC_50_	CC_50_	SI
**BHK**	1.9 ± 0.2	360 ± 43	184	5.3 ± 0.8	177 ± 22	33	7.3 ± 0.3	ND	NA
**SH-sy5y**	1.1 ± 0.2	421 ± 34	384	2.9 ± 0.4	300 ± 21	103	9.8 ± 1.2	ND	NA
**Huh-7**	0.41 ± 0.04	381 ± 25	1191	6.9 ± 0.8	284 ± 12	41	8.2 ± 0.3	ND	NA
**Vero**	>50	512 ± 43	NA	5.5 ± 0.04	321 ± 41	58	9.1 ± 0.2	ND	NA

^*^Values are expressed as U/ml, ND – Not determined, NA – Not Applicable.

**Table 2:** Antiviral activity and cytotoxicity of sofosbuvir at an MOI of 0.5.

	Sofosbuvir			Ribavirin			IFN-alpha*		
	EC_50_	CC_50_	SI	EC_50_	CC_50_	SI	EC_50_	CC_50_	SI
**BHK**	1.7 ± 0.1	360 ± 43	212	3.1 ± 0.6	177 ± 22	57	2.8 ± 0.3	ND	NA
**SH-sy5y**	0.65 ± 0.08	421 ± 34	648	1.2 ± 0.2	300 ± 21	31	4.9 ± 0.6	ND	NA
**Huh-7**	0.12 ± 0.03	381 ± 29	929	3.8 ± 0.04	284 ± 19	23	3.4 ± 0.1	ND	NA
**Vero**	>50	512	NA	3.3 ± 0.08	321 ± 56	97	8.3 ± 0.2	ND	NA

^*^Values are expressed as U/ml, ND – Not determined, NA – Not Applicable.

**Table 3:** Mutations in the ZIKV sequence from viruses propagated in cells treated with 2 μM sofosbuvir.

Mutation Type	Number of mutations						Frequency per 1000 nucleotides€					
	Huh-7		NSC		Organoid		Huh-7		NSC		Organoid	
	Control	SFV	Control	SFV	Control	SFV	Control	SFV	Control	SFV	Control	SFV
All Mutations	11	19	13	22	15	27	0.210	0.363	0.248	0.420	0.287	0.516
Transitions	8	15	7	16	12	22	0.153	0.287*	0.134	0.306*	0.229	0.420*
A-to-G	2	13	3	12	4	18	0.038	0.248*	0.057	0.229*	0.076	0.344*
Transversions	3	4	6	6	3	5	0.057	0.076	0.115	0.115	0.057	0.096

^€^With respect to 52347 nucleotides analyzed, SFV – sofosbuvir, ^*^P < 0.05 SFV vs control.
