# Corrigendum: The clinically approved antiviral drug sofosbuvir inhibits Zika virus replication

**DOI:** 10.1038/srep46772

**Published:** 2017-04-24

**Authors:** Carolina Q. Sacramento, Gabrielle R. de Melo, Caroline S. de Freitas, Natasha Rocha, Lucas Villas Bôas Hoelz, Milene Miranda, Natalia Fintelman-Rodrigues, Andressa Marttorelli, André C. Ferreira, Giselle Barbosa-Lima, Juliana L. Abrantes, Yasmine Rangel Vieira, Mônica M. Bastos, Eduardo de Mello Volotão, Estevão Portela Nunes, Diogo A. Tschoeke, Luciana Leomil, Erick Correia Loiola, Pablo Trindade, Stevens K. Rehen, Fernando A. Bozza, Patrícia T. Bozza, Nubia Boechat, Fabiano L. Thompson, Ana M. B. de Filippis, Karin Brüning, Thiago Moreno L. Souza

Scientific Reports 7: Article number: 4092010.1038/srep40920; published online 01 18 2017; updated on 04 24 2017

The Competing Financial Interests section in this Article is incorrect and should read:

“Dr. Karin Brüning is a member of the BMK consortium, able to produce sofosbuvir”.

