# Supplementary material for: The clinically approved antiviral drug sofosbuvir inhibits Zika virus replication
Source: Sci Rep. 2017 Jan 18;7:40920. doi: 10.1038/srep40920 (PMC5241873; doi:10.1038/srep40920)
Supplement: Supplementary Information [file srep40920-s1.pdf]

Supplementary information

The clinically approved antiviral drug sofosbuvir inhibits Zika virus replication

Carolina Q. Sacramento[#], Gabrielle R. de Melo[#], Caroline S. de Freitas, Natasha Rocha, Lucas Villas Bôas Hoelz, Milene Miranda, Natalia Fintelman-Rodrigues, Andressa Marttorelli, André C. Ferreira, Giselle Barbosa-Lima, Juliana L. Abrantes, Yasmine Rangel Vieira, Mônica M. Bastos, Eduardo de Mello Volotão, Estevão Portela Nunes, Diogo A. Tschoeke, Luciana Leomil, Erick Correia Loiola, Pablo Trindade, Stevens K. Rehen, Fernando A. Bozza, Patrícia T. Bozza, Nubia Boechat, Fabiano L. Thompson, Ana M. B. de Filippis, Karin Brüning and Thiago Moreno L. Souza

# - These authors contributed equally to this work

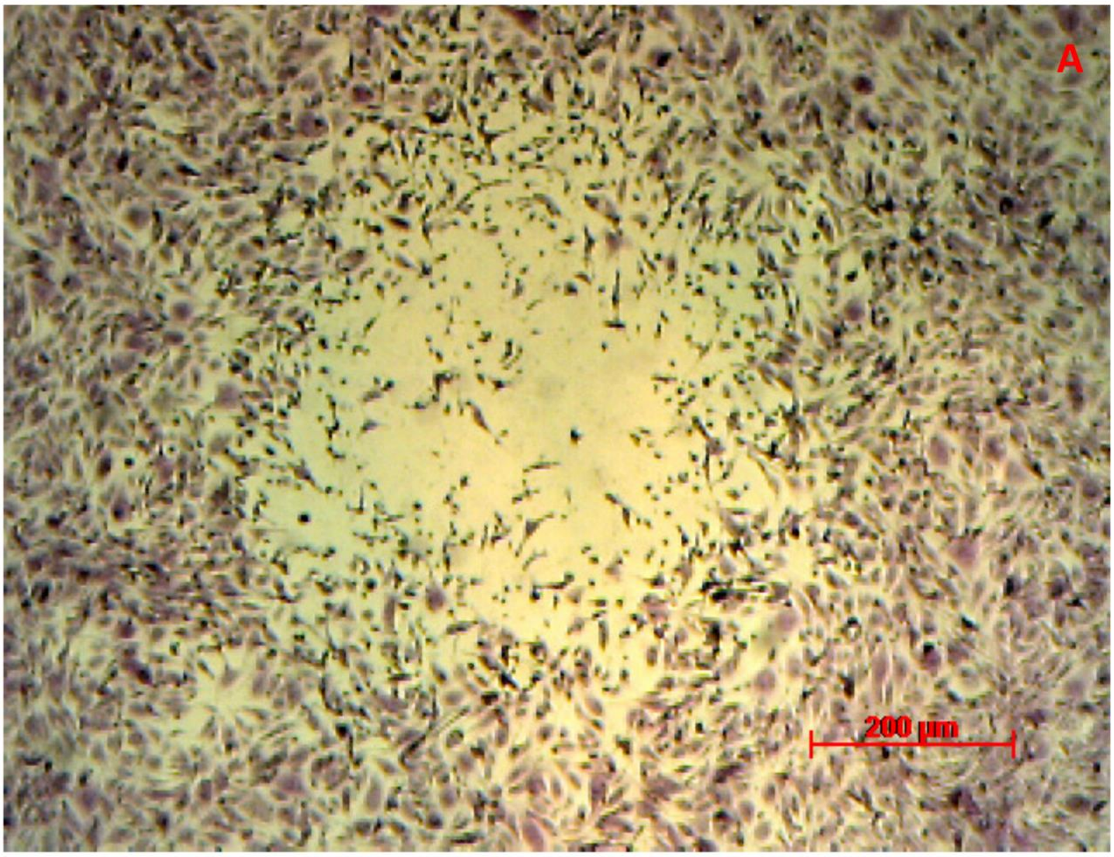
A
200 µm

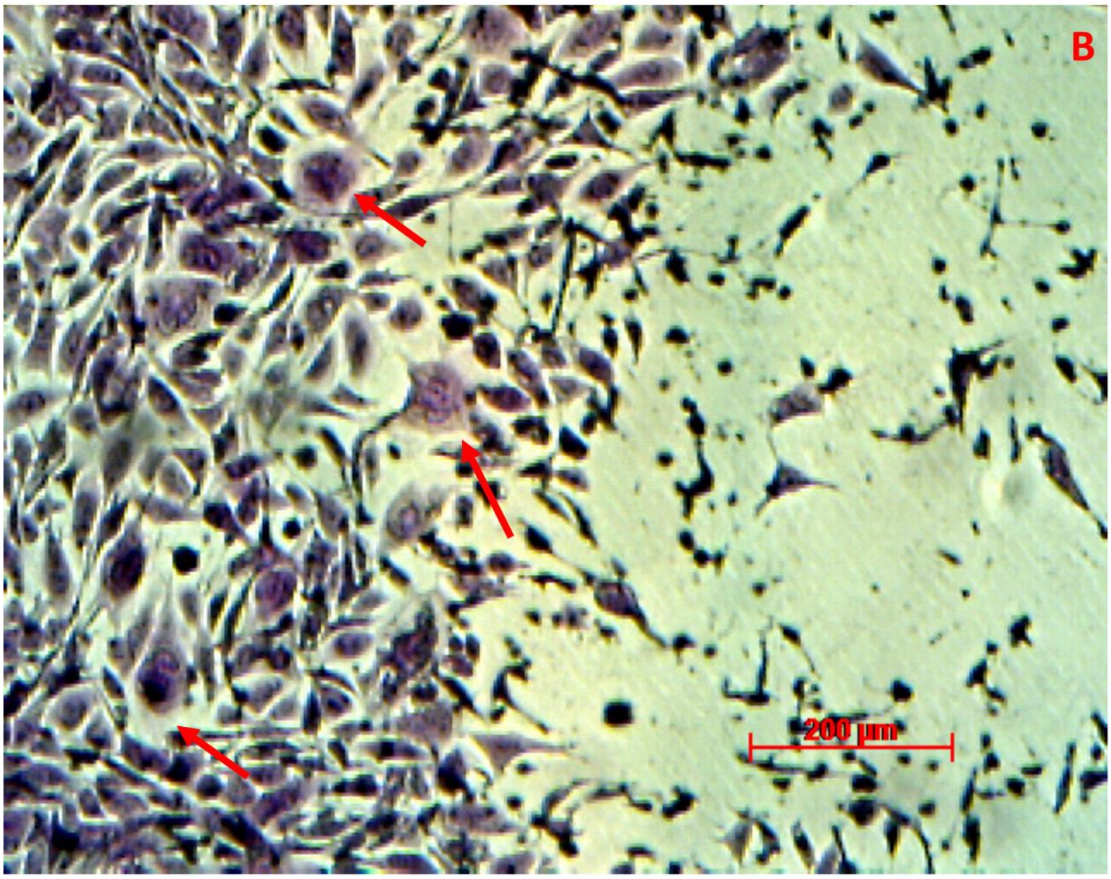
B
200 µm

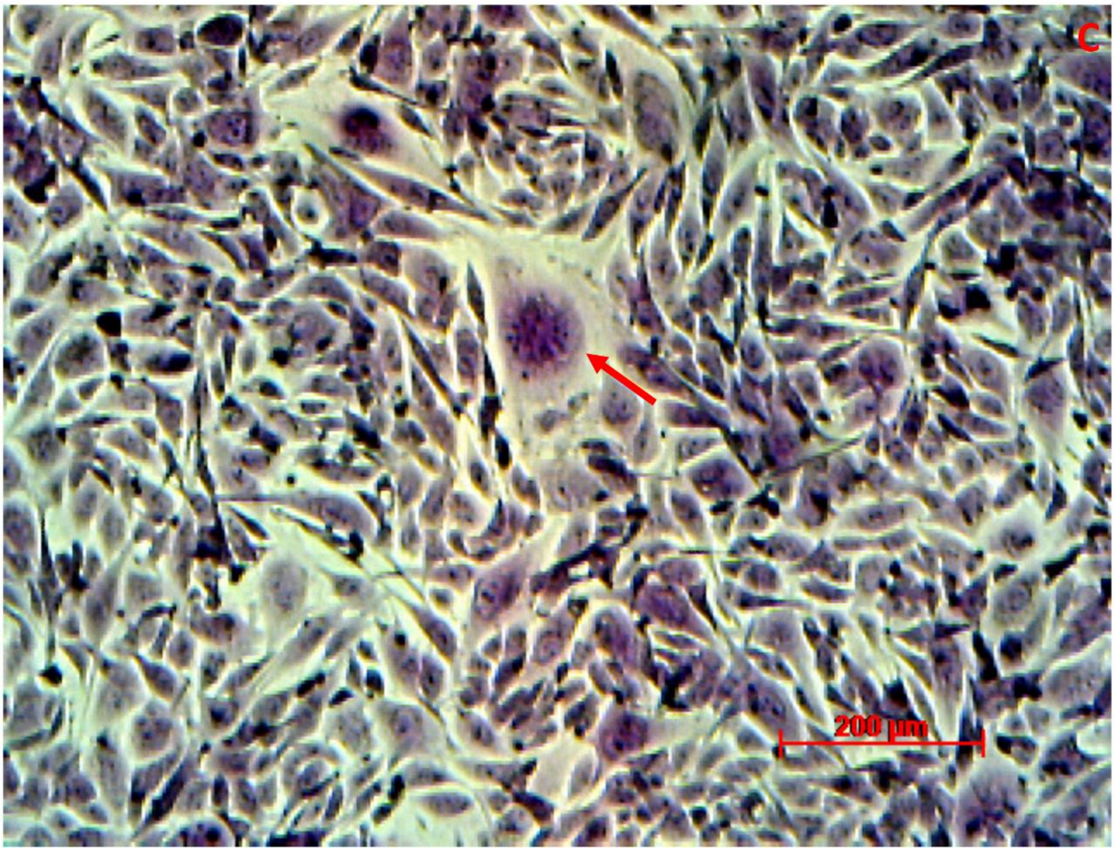
C
200 µm

Figure S1 – Plaque assay for ZIKV. Monolayers of BHK-21 cells were infected for 1 h at 37 °C. Thereafter, the viral particles were removed by washing with PBS, and the wells were covered with overlay medium containing 1 % FBS. At 5 days post infection, plaques were fixed and stained with crystal violet. (A) A representative plaque-forming unit (PFU) is presented at 40x magnification. (B) The PFU and adjacent cellular monolayer is presented at 100x magnification; some of these cells exhibit ZIKV-induced cytopathic effects (CPEs), and three examples are highlighted by the red arrows. (C) A representative closer view of ZIKV-induced CPEs (red arrow).

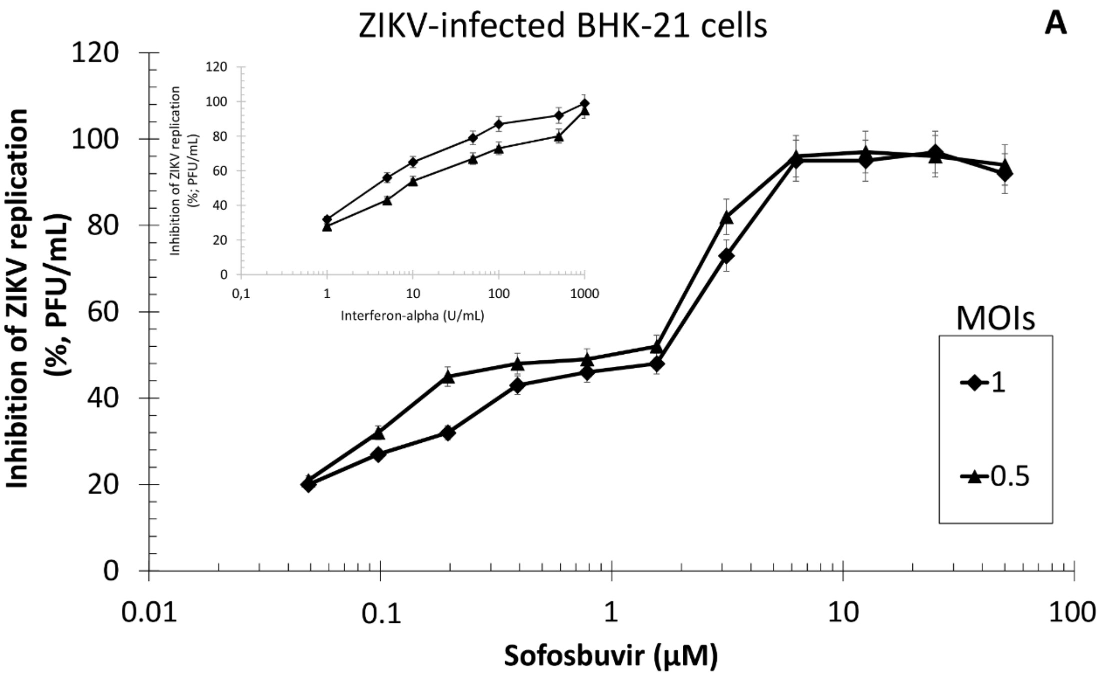
ZIKV-infected BHK-21 cells
A
Inhibition of ZIKV replication (%, PFU/mL)
Sofosbuvir (µM)
Inhibition of ZIKV replication (%; PFU/mL)
Interferon-alpha (U/mL)
MOIs
1
0.5

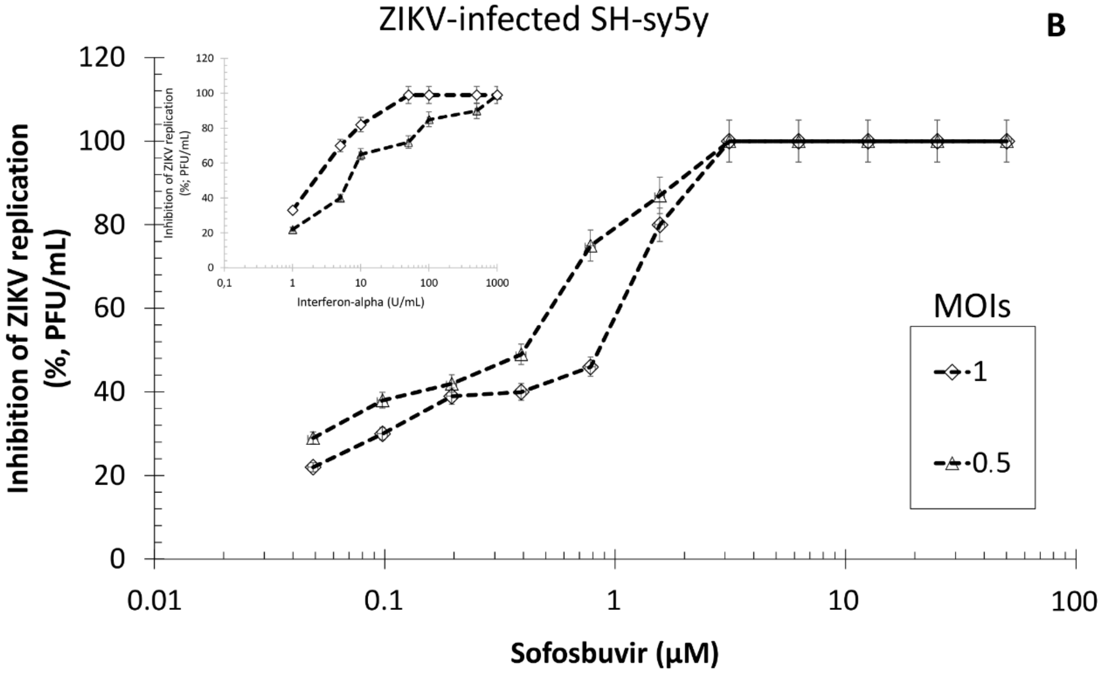
ZIKV-infected SH-sy5y
B
Inhibition of ZIKV replication (%, PFU/mL)
Sofosbuvir (µM)
Inhibition of ZIKV replication (%; PFU/mL)
Interferon-alpha (U/mL)
MOIs
1
0.5

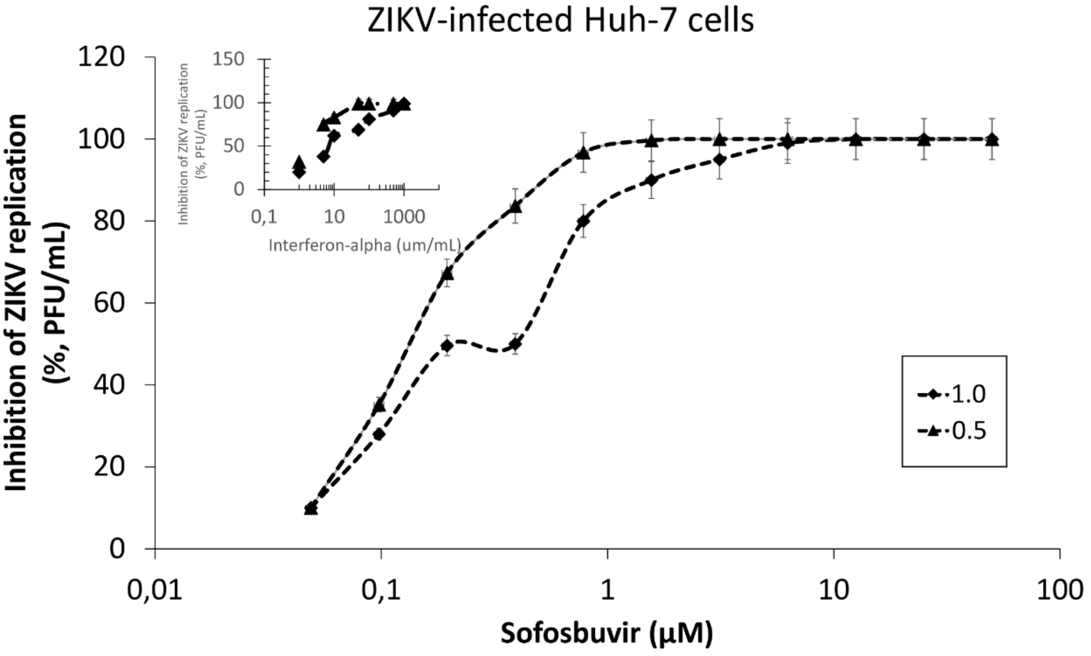

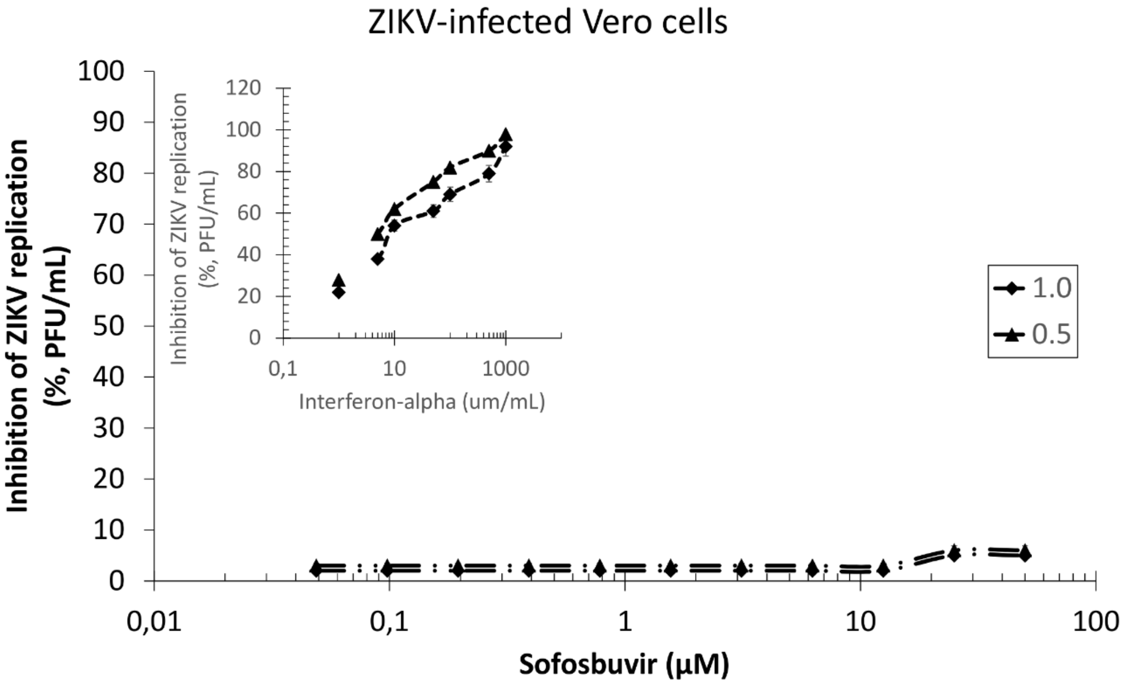

Figure S2 – The antiviral activity of sofosbuvir against ZIKV. BHK-21 (A), SH-Sy5y (B), Huh-7 (C) or Vero (D) cells were infected with ZIKV at the indicated MOIs and exposed to various concentrations of sofosbuvir or IFN-alpha (inset), and viral replication was measured by a plaque-forming assay after 24 h of infection. The data represent means ± SEM of five independent experiments.

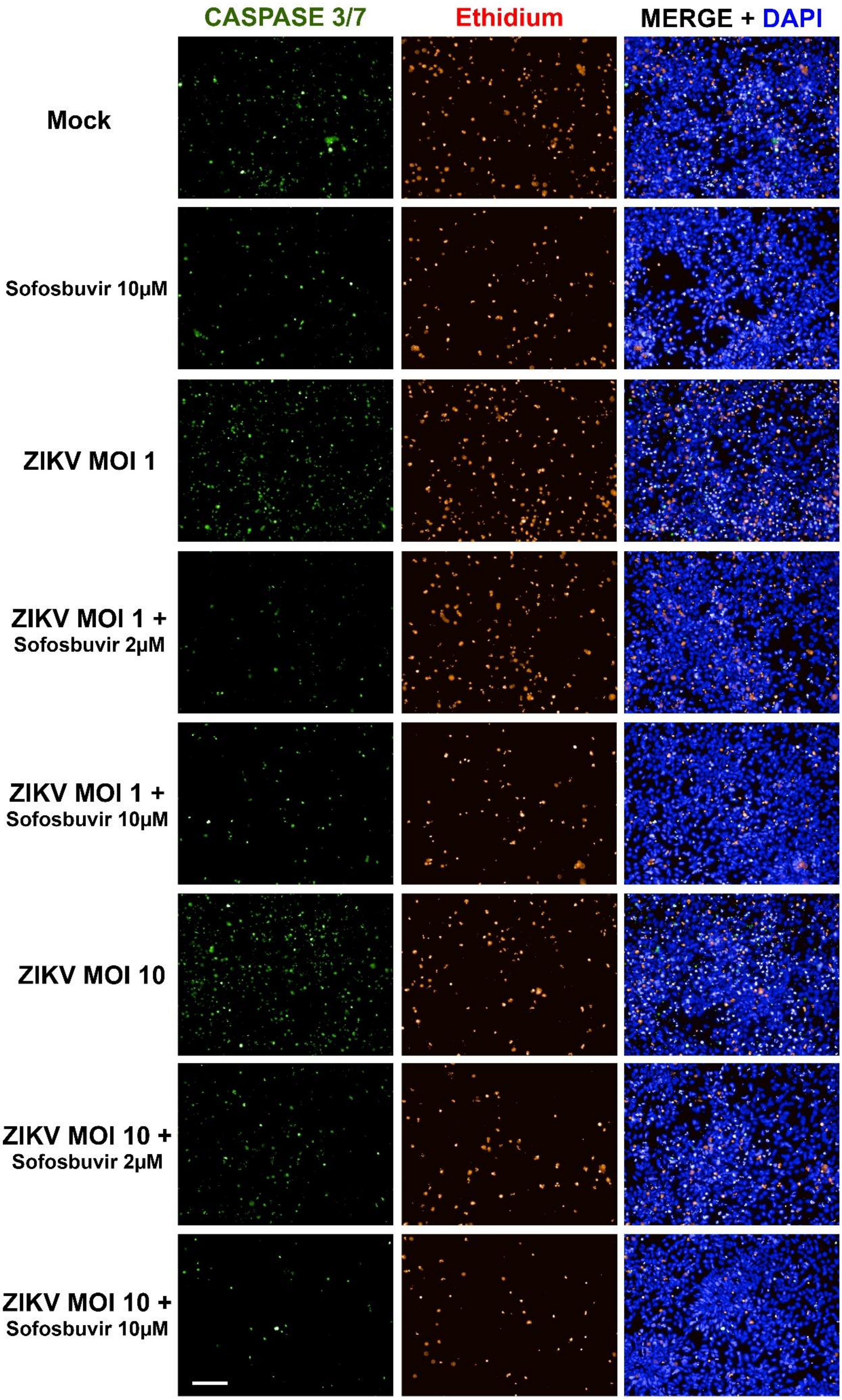
CASPASE 3/7
Ethidium
MERGE + DAPI
Mock
Sofosbuvir 10μM
ZIKV MOI 1
ZIKV MOI 1 +
Sofosbuvir 2μM
ZIKV MOI 1 +
Sofosbuvir 10μM
ZIKV MOI 10
ZIKV MOI 10 +
Sofosbuvir 2μM
ZIKV MOI 10 +
Sofosbuvir 10μM

Figure S3 – Sofosbuvir inhibits ZIKV-induced caspase-3/7 activation in human iPS cell-derived NSCs. Four days after infection, NSCs were labeled for activated caspase-3/7 (left panels) and cell permeability (middle panels), and the images were merged with images of DAPI staining (right panels). Bar = 100 µM

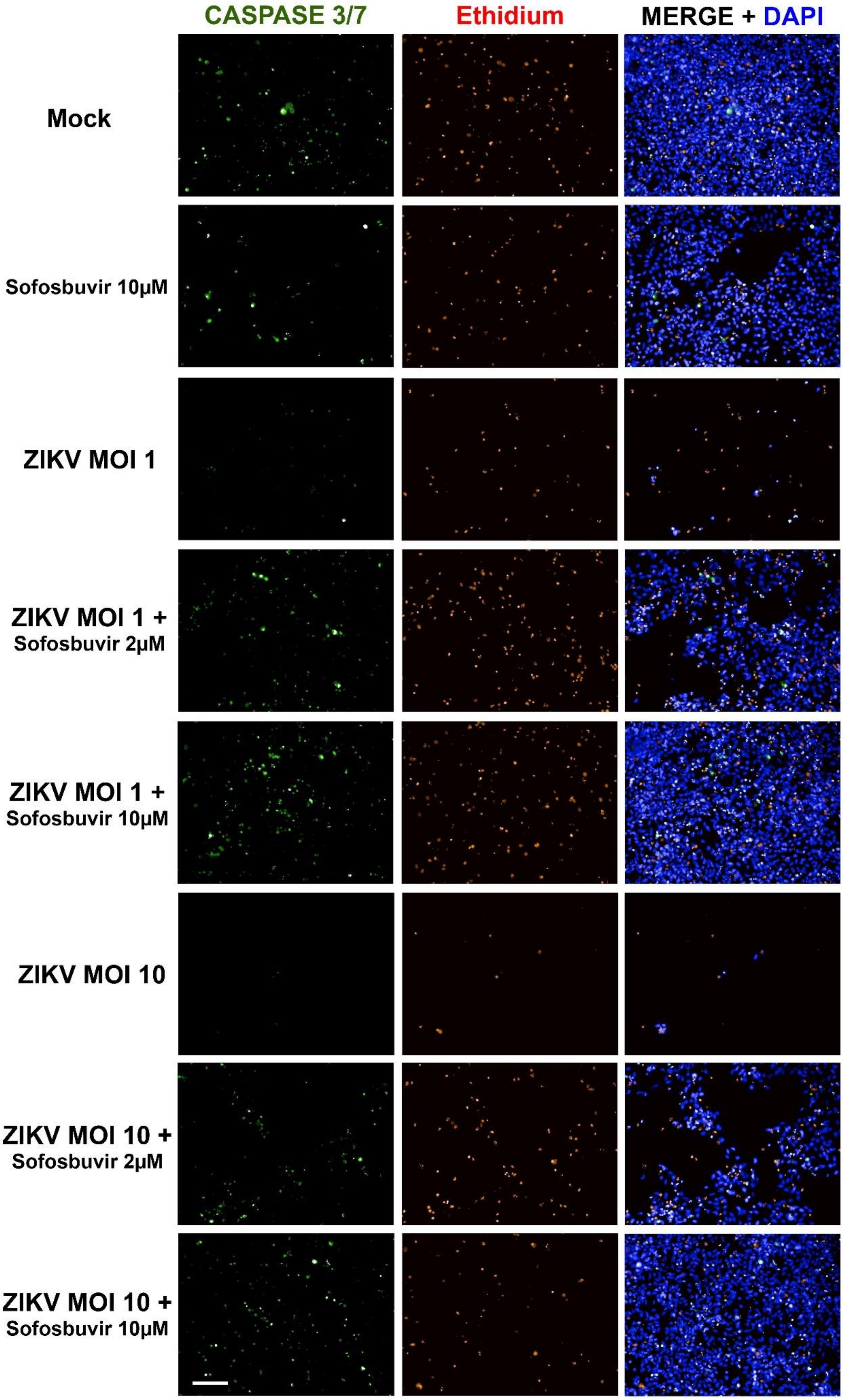
CASPASE 3/7
Ethidium
MERGE + DAPI
Mock
Sofosbuvir 10μM
ZIKV MOI 1
ZIKV MOI 1 +
Sofosbuvir 2μM
ZIKV MOI 1 +
Sofosbuvir 10μM
ZIKV MOI 10
ZIKV MOI 10 +
Sofosbuvir 2μM
ZIKV MOI 10 +
Sofosbuvir 10μM

Figure S4 – Sofosbuvir protects human iPS cell-derived NSCs from ZIKV-induced cell death. Eight days after infection, NSCs were labeled for activated caspase-3/7 (left panels) and cell permeability (middle panels), and the images were merged with DAPI staining (right panels). Bar = 100 µM.
